# Bi-directional coupling in strain-mediated multiferroic heterostructures with magnetic domains and domain wall motion

**DOI:** 10.1038/s41598-018-23020-2

**Published:** 2018-03-26

**Authors:** Zhuyun Xiao, Roberto Lo Conte, Cai Chen, Cheng-Yen Liang, Abdon Sepulveda, Jeffrey Bokor, Gregory P. Carman, Robert N. Candler

**Affiliations:** 1 Department of Electrical and Computer Engineering, University of California, Los Angeles, California 90095 USA; 2 Department of Electrical Engineering and Computer Sciences, University of California, Berkeley, California 94720 USA; 3 Department of Mechanical and Aerospace Engineering, University of California, Los Angeles, California 90095 USA; 4 California NanoSystems Institute, Los Angeles, California 90095 USA

## Abstract

Strain-coupled multiferroic heterostructures provide a path to energy-efficient, voltage-controlled magnetic nanoscale devices, a region where current-based methods of magnetic control suffer from Ohmic dissipation. Growing interest in highly magnetoelastic materials, such as Terfenol-D, prompts a more accurate understanding of their magnetization behavior. To address this need, we simulate the strain-induced magnetization change with two modeling methods: the commonly used unidirectional model and the recently developed bidirectional model. Unidirectional models account for magnetoelastic effects only, while bidirectional models account for both magnetoelastic and magnetostrictive effects. We found unidirectional models are on par with bidirectional models when describing the magnetic behavior in weakly magnetoelastic materials (e.g., Nickel), but the two models deviate when highly magnetoelastic materials (e.g., Terfenol-D) are introduced. These results suggest that magnetostrictive feedback is critical for modeling highly magnetoelastic materials, as opposed to weaker magnetoelastic materials, where we observe only minor differences between the two methods’ outputs. To our best knowledge, this work represents the first comparison of unidirectional and bidirectional modeling in composite multiferroic systems, demonstrating that back-coupling of magnetization to strain can inhibit formation and rotation of magnetic states, highlighting the need to revisit the assumption that unidirectional modeling always captures the necessary physics in strain-mediated multiferroics.

## Introduction

Controlled magnetization motion, including domain-wall (DW) and domain state rotation, in miniaturized multiferroic heterostructures creates the possibility of new types of devices in a range of applications, including memory^1^, logic devices^2,3^ and nanoscale sensors^4^/actuators^5^. Previous micro/nanoscale DW-based devices used either external magnetic field or current-based methods to manipulate DWs^6^. However, the external magnetic field approach fails to realize localized magnetic state switching, and current-based approach suffers from power consumption and thermal management issues due to Joule heating^7^. Alternative approaches to control DW and magnetization motion at micro/nanoscale are thus required to address these issues.

**Figure 1:**
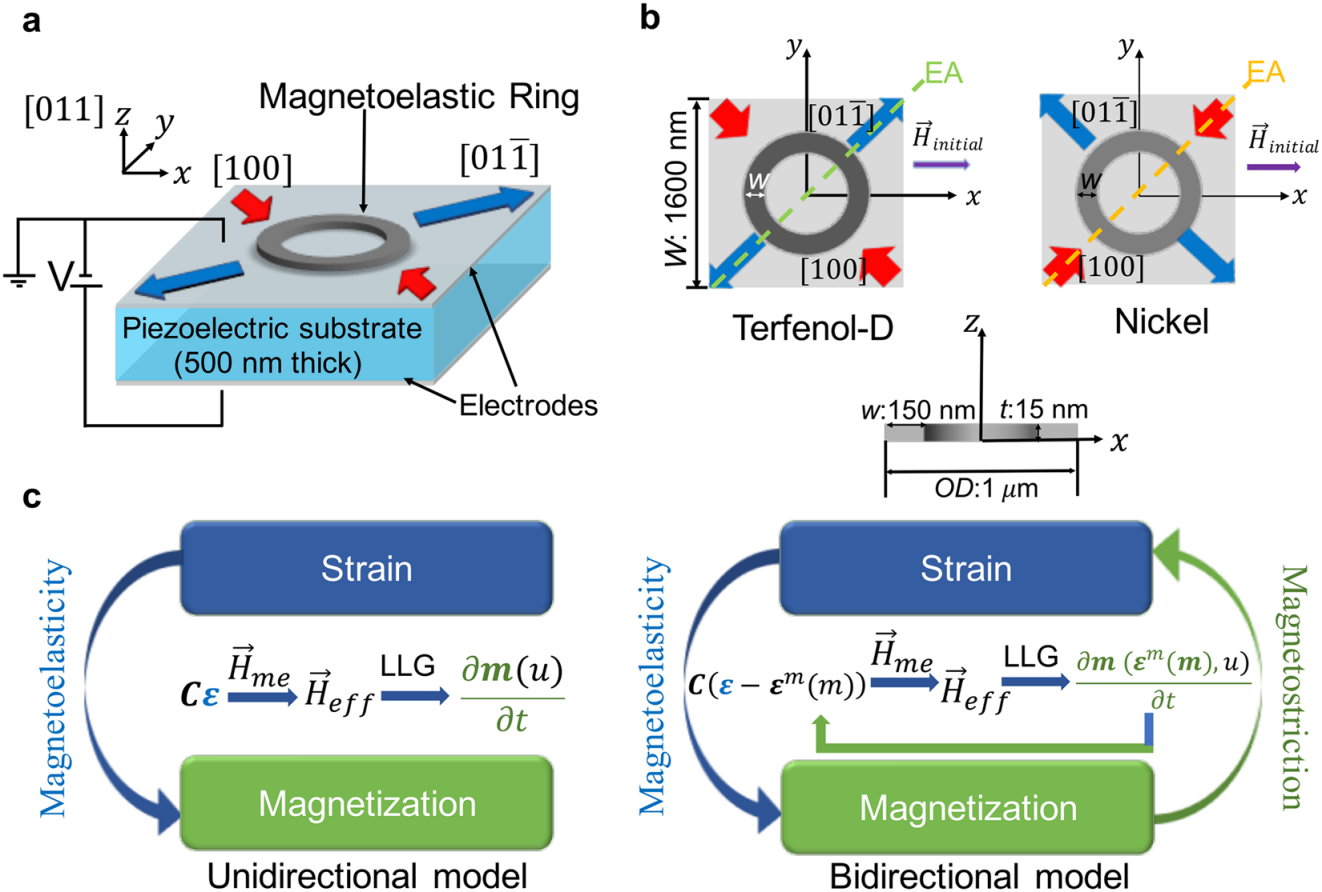
Schematic illustrations of (**a**) setup for the electrical-field controlled strain-mediated rotation of magnetic domain states in a ferromagnetic ring on top of piezoelectric substrate PMN-PT with a 500 µm thickness. (**b**) Top view and cross section view of the magnetoelastic rings (Terfenol-D and Ni) with outer diameter (*OD*) of 1 μm, width (*w*) of 150 nm and thickness (*t*) of 15 nm. Illustration of the initialization field ${\overrightarrow{H}}_{initial}\,\,$with respect to the crystal orientations in PMN-PT for both Terfenol-D and Ni rings, and the tensile and compressive response along corresponding directions. Substrate width is 1600 nm. EA indicates the strain-induced magnetic easy-axis due to magneto-elastic coupling. (**c**) Description of the two simulation approaches: the unidirectional model only tracks inverse magnetostrictive effect; while the bidirectional model considers both the magnetostrictive and inverse magnetostrictive effects.

The use of electric fields to control magnetization through multiferroic coupling subsequently emerged as an alternative due to the favorable scaling of electric fields as compared to currents^8–14^. Strain-coupled multiferroic heterostructures, which consist of a non-magnetic\ferromagnetic bilayer, have been investigated as an alternative pathway to achieve energy efficient magnetization control at room temperature^2,15,16^. In particular, strain-coupled multiferroics have a ferromagnetic (FM) layer and ferroelectric/piezoelectric substrate (strain-mediated approach) where the strain in the piezoelectric layer alters the magnetic anisotropy in the FM layer (Fig. 1a and b)^17,18^.

Manipulation of magnetization via the strain-based approach has already been demonstrated experimentally in Ni^10,19–22^, CoFeB^23^, FeGa^24^, Fe and CoFe^9^ on piezoelectric substrates. Increasing interest in highly magnetoelastic materials, such as Terfenol-D (Tb_x_Dy_1−x_Fe_2_, x = 0.3) with magnetostriction saturation ${\lambda }_{s}$ = $1200\times {10}^{-6}$ ^25^, creates the need for thorough understanding of the magnetization behavior inside these materials owing to their potential for enhanced strain-mediated DW rotation^26–29^. As a side note, maximizing magnetostriction is often a desirable pursuit, although there are cases where there are nuanced tradeoffs and maximum magnetostriction is not necessarily a standalone objective. Much of the prior work that uses unidirectional (UD) model has produced comparable results to the experimental observations, even though strain induced by the change in magnetization is generally ignored^30–33^. A UD model only tracks magnetoelastic coupling in one direction, i.e. a static electric-field produces a strain which changes the magnetic anisotropy but does not feedback to the initial equilibrium strain state. However, these results usually focus on weakly magnetoelastic materials, including pure transition metal ferromagnets such as Ni (${\lambda }_{s}$ = $-33\times {10}^{-6})$^32^. For materials such as Terfenol-D, with a much larger saturation magnetostriction, previous UD models may no longer be adequate for describing the system, and thus require a more rigorous approach. In the aforementioned multiferroic heterostructures (Fig. 1a), not only will the strain in the piezoelectric substrate influence the magnetic anisotropy in the magnetic film owing to the inverse magnetostrictive effect (Villari effect)^34^, but also the change in magnetization in the film will in turn feedback to the piezoelectric material via the magnetostrictive effect. A bidirectional (BD) model can fully account for such an interaction. To understand the differences between the two approaches, we compare the simulated magnetic domain and DW rotations in both Ni and Terfenol-D disks and rings for different voltage-induced strains.

In this report, we first investigate the initialized DW states in magnetic structures at equilibrium using both the UD and BD modeling approaches. We consider two common elements in multiferroics, rings and disks^35,36^, due to their radial symmetry and smooth sidewalls. We then apply strain to the structures and study the strain-induced domain motion predicted by the two approaches, with the resulting magnetic states compared and contrasted. The simulation results show that for weakly magnetoelastic materials (e.g. Ni, Co and CoFeB)^10,33,35,37^ with applied strain, the influence of magnetostriction is likely negligible, and UD models are sufficient. However, the transition to highly magnetoelastic materials (e.g. Terfenol-D) requires a more accurate accounting of the coupling, and BD models should therefore be considered. We also report classifications of initialized magnetization configurations in Terfenol-D rings with different dimensions and present a phase diagram of equilibrium magnetic domain states (see Supplementary Note S1), which lays the groundwork for properly designing Terfenol-D ring systems. The focus of this study is the behavior of the nucleated magnetic DWs (see Supplementary Note S1) or magnetic domains in response to such strain. This is due to the fact that the localized magnetic stray field from the DWs and domains can be utilized in actual technological applications, such as nanoparticle manipulation in microfluidic environments^38,39^.

## Modeling Setup

The schematic of the modeling setup for electric-field controlled, strain-mediated DW rotation in a ring is illustrated in Fig. 1a. A piezoelectric substrate, [Pb(Mg_1/3_Nb_2/3_)O_3_]_0.66_–[PbTiO_3_]_0.34_ (PMN–PT), with a size of 1600 nm $\times$ 1600 nm $\times$ 500 nm (thickness) is placed underneath a magnetoelastic ring or disk. The bottom surface of the PMN-PT is clamped with no displacement, and the four sides of the PMN-PT substrate are also clamped (see Supplementary Note S2)^40^. Simulations of varying substrate sizes verified that the chosen substrate is sufficiently large to accurately model the strain in the magnetic structures. The ring dimension (Fig. 1b) is chosen as 1000 nm outer diameter (*OD)* with 150 nm width (*w*) and 15 nm thickness (*t*) so that the initialized stable state before strain application is an onion state with transverse DWs^19^, with a large total energy density (see Supplementary Note S1 for a list of geometry-dependent initial states). The disk dimension is chosen with 1000 nm in diameter and 15 nm in thickness. The choice of the 15 nm thickness for the magnetic structures ensures that the strain is effectively transferred across the entire magnetic layer thickness^41^.

The magnetic states of the ring and disk are initially set in equilibrium states, representing the remanent state after removal of an external magnetic field that initially saturated the state in the +*x* direction. Once the field is removed, the magnetization falls into a magnetically-relaxed state due to minimization of the total energy^19,36^, including demagnetization energy (shape anisotropy energy) and exchange energy. For a ring, this step generates the nucleation of two DWs in diametrically opposite position.

After the initialization process, an electric field is applied through the thickness of the PMN-PT [011] substrate, which is in a pre-poled ferroelectric state, with polarization pointing up (or down). In such strain-mediated multiferroic approach, a voltage applied to the PMN-PT substrate induces anisotropic strain in the magnetoelastic structures. This results in a mechanical compressive strain along the [100] direction, and tensile strain along the [$01\bar{1}$] direction of the piezoelectric substrate (see Supplementary Note S2). The electrically-induced strain is then transferred into the ring and the disk. Magnetostrictive materials usually exhibit complicated domain structures to minimize total free energy, which is determined by the competition among exchange energy, demagnetization energy and magneto-elastic energy^40,42^. With the presence of applied strain, reorientation of the domain states take place because of such competition.

In a Terfenol-D ring, as shown in Fig. 1b, the transferred mechanical strain tends to orient the DWs toward the *tensile* strain axis direction [$01\bar{1}$] in PMN-PT (45° from the +*x* axis) due to a *positive* magnetostriction effect^43,44^. On the other hand, for a Ni ring on PMN-PT (see Fig. 1b), DWs tend to rotate toward the *compressive* strain direction [100] due to the *negative* saturation magnetostriction of Ni. Therefore, different from the Terfenol-D ring system setup, for the piezoelectric substrate underneath Ni ring, compressive strain is induced along 45° from the +*x* axis (also [100] direction), and tensile strain along −45° to the +*x* axis (also [$01\bar{1}$] direction) to produce DW rotation in the same counter clockwise direction as that in Terfenol-D rings to make clear comparisons (see Fig. 1b for more details).

In actual ring devices, the strain is not uniformly transferred from the piezoelectric to the ferromagnetic layer, resulting in a non-uniform magneto-elastic energy density through the ring width and thickness. Non-uniformity of the strain arises from different mechanisms, such as shear lag effects of the strain and anisotropy in the piezoelectric substrate^40^. Due to the non-uniform strain distribution in the system, mapping of the time-dependent inhomogeneous strain states is required in the modeling.

For the simulation, we do not explicitly take into account thermal fluctuations and induced noise to the system, although we do use room temperature values for material parameters such as exchange constant A_ex_, the saturation magnetization Ms, magnetoelastic coupling coefficients B_1_, B_2_ and magentostriction constants ${\lambda }_{100}$, ${\lambda }_{111}$. Since no intrinsic and extrinsic defects were introduced^45^ to the geometries, the discrepancies between the domain movement in the system at 0 K and elevated temperatures (e.g., T = 300 K) will be insignificant^46^.

## Computational Details

We simulated the strain-induced magnetization change using both the BD and UD models. For both models, the micromagnetic and elastodynamic partial differential equations (PDEs) are implemented in the weak form and are solved using the finite element method. However, the two models differ because the BD model, differently from the UD model, incorporates stress induced via magnetostriction, iterating between stress-induced changes in magnetization, and magnetization-induced stress until a solution is found.

From the magnetic point of view, the time evolution of the normalized magnetization ${\boldsymbol{m}}$ ($|{\boldsymbol{m}}|=1$) is determined by the micromagnetic relation that satisfies the Landau-Lifshitz-Gilbert equation (LLG). LLG describes the precessional dynamics and relaxation of the magnetization vector:

$$\frac{\partial {\boldsymbol{m}}}{\partial t}=-{\mu }_{0}\gamma ({\boldsymbol{m}}\times {{\boldsymbol{H}}}_{{\boldsymbol{eff}}})+\alpha ({\boldsymbol{m}}\times \frac{\partial {\boldsymbol{m}}}{\partial t}),$$

where ${\mu }_{0}$ is the vacuum permeability, γ is the gyromagnetic ratio and $\alpha \,\,$is the Gilbert damping constant. For Ni, the experimentally measured $\alpha$ is 0.038^47,48^, and for Terfenol-D it is 0.06 ± 0.02^49^. In our simulations, the major goal is to compare the final static state after strain is applied, so ${\rm{\alpha }}$ is set to 0.5 to achieve equilibrium in a reasonable calculation time. The damping factor is expected to affect the magnetization dynamics, including the speed with which the DW/domain will move and the time that will take for the magnetization to reach its stable state. However, it is not expected to affect the final state itself, which is determined by the minimization of total free energy^40^. In our study, we induce a uniaxial magnetic anisotropy which will induce the magnetization to realign. This reorientation is expected to be fully deterministic if a magnetization rotation of an angle smaller than 90° is induced, which is the case in this study. Accordingly, we do not expect the larger damping factor to influence at all the final magnetic state induced by the applied strain via magneto-elastic coupling. The effective magnetic field ${{\rm{H}}}_{\mathrm{eff}}\,$is defined as ${{\rm{H}}}_{{\rm{eff}}}=-\frac{1}{{{\rm{\mu }}}_{0}{{\rm{M}}}_{{\rm{s}}}}\frac{\partial {{\rm{E}}}_{{\rm{tot}}}}{\partial {\rm{m}}}$, where ${{\rm{E}}}_{{\rm{tot}}}\,\,$is the total energy density and ${{\rm{M}}}_{{\rm{s}}}$ is the saturation magnetization^50^. In our model, ${{\rm{H}}}_{{\rm{eff}}}\,\,$is expressed as the summation of the external field $({{\rm{H}}}_{{\rm{ext}}})$, exchange field $({{\rm{H}}}_{{\rm{ex}}}),$ demagnetization field $({{\rm{H}}}_{{\rm{d}}})\,\,$and magnetoelastic field $({{\rm{H}}}_{{\rm{me}}})$^40,51^. Among these fields, the magnetoelastic field ${{\rm{H}}}_{{\rm{me}}}({\rm{m}},\,{{\rm{\varepsilon }}}^{{\rm{tot}}})$ depends on both ${\rm{m}}$ and the total strain ${{\rm{\varepsilon }}}^{{\rm{tot}}}$. Solving these equations using the finite element method allows us to determine the final magnetization of the magnetic structure.

From the mechanical point of view, the displacement field ${\boldsymbol{u}}$ obeys the elastodynamic equation $\rho \frac{{\partial }^{2}{\boldsymbol{u}}}{\partial {t}^{2}}=\nabla \cdot {\boldsymbol{\sigma }}$, where ${\rm{\rho }}$ and ${\boldsymbol{\sigma }}$ denote the volumetric density and the stress tensor, respectively^3,40^. Thus, the constitutive relation between the stress tensor ${\boldsymbol{\sigma }}$ and the elastic strain tensor ${{\boldsymbol{\varepsilon }}}^{{\boldsymbol{el}}}$ can be expressed as ${\boldsymbol{\sigma }}={\boldsymbol{C}}{{\boldsymbol{\varepsilon }}}^{{\boldsymbol{el}}}$, where ${\boldsymbol{C}}$ is the elastic stiffness tensor. In magnetoelastic materials that are also cubic crystals, the magnetoelastic strain tensor ${{\boldsymbol{\varepsilon }}}^{m}$ is induced by ${\boldsymbol{m}}$:

$${{\rm{\varepsilon }}}_{ii}^{m}=\frac{3}{2}{\lambda }_{100}({m}_{i}^{2}-\frac{1}{3})\,{\rm{and}}\,{\varepsilon }_{ij}^{m}=\frac{3}{2}{\lambda }_{111}{m}_{i}{m}_{j}(i\ne j),$$

where ${\lambda }_{100}$ and ${\lambda }_{111}$ represent the magnetostriction constants in 〈100〉 and 〈111〉 directions, respectively. ${{\boldsymbol{\varepsilon }}}^{m}$ also contributes to ${{\boldsymbol{\varepsilon }}}^{tot}$, namely ${{\boldsymbol{\varepsilon }}}^{tot}={{\boldsymbol{\varepsilon }}}^{el}+{{\boldsymbol{\varepsilon }}}^{m}.$

The major difference between the BD and UD models originates from the way in which the strain is being treated. In the conventional UD model, elastic stain ${\varepsilon }^{el}$ is assumed to be the only strain contributing to the magnetoelastic effects, and is thus equivalent to the total strain ${\varepsilon }^{tot}$ (see Fig. 1c, left). Therefore, elastodynamics and micromagnetics are not fully coupled, as the magnetization is calculated in the following steps: a) ${{\boldsymbol{\varepsilon }}}^{{\boldsymbol{tot}}}$, which equates to ${{\boldsymbol{\varepsilon }}}^{{\boldsymbol{el}}}$ in UD models, is calculated first by solving the elastodynamic equation, b) ${{\boldsymbol{\varepsilon }}}^{{\boldsymbol{tot}}}$ is incorporated into the LLG equation via ${{\boldsymbol{H}}}_{me}$ to calculate ${\boldsymbol{m}}$ in the magnetoelastic structure.

On the other hand, in the BD model, the total strain ${{\boldsymbol{\varepsilon }}}^{{\boldsymbol{tot}}}$ takes into account contributions from both the linear elastic strain and the magnetic strain. It solves the intrinsically coupled PDEs simultaneously: a) ${{\boldsymbol{\varepsilon }}}^{{\boldsymbol{tot}}}$, which equates to ${{\boldsymbol{\varepsilon }}}^{{\boldsymbol{el}}}$**+**${{\boldsymbol{\varepsilon }}}^{m}$, is calculated from the elastodynamic equation, b) *ε*^*tot*^ is incorporated into the LLG equation via ***H***_*me*_ to calculate ${\boldsymbol{m}}$ as well as ${{\boldsymbol{\varepsilon }}}^{m}$, and c) the generated magnetic state is fed back into the elastodynamic equation and the above steps are repeated until reaching convergence. As shown in Fig. 1c (right), magnetization change causes change in strain by affecting ${{\boldsymbol{\varepsilon }}}^{{\boldsymbol{m}}}({\boldsymbol{m}})$, and hence ${{\boldsymbol{H}}}_{me}$. Consequently, the time-dependent distribution of magnetization vectors ${\boldsymbol{m}}\,({{\boldsymbol{\varepsilon }}}^{m}({\boldsymbol{m}}),\,u,\,t)$ in the magnetoelastic structure, which responds to both displacement field ***u*** and recurring changes in effective strain imposed by ${{\boldsymbol{\varepsilon }}}^{m}({\boldsymbol{m}})$, continue to modify ${{\boldsymbol{\varepsilon }}}^{{\boldsymbol{m}}}({\boldsymbol{m}})$ and thus ${{\boldsymbol{H}}}_{me}$ (as illustrated by the green arrow in Fig. 1c, right). This bidirectional model captures the bilateral communication/interaction between strain and magnetization via both Villari effect and magnetostrictive effect (see Fig. 1c).

By comparison, the BD model more fully captures the physics in the coupled magnetoelastic system. The decoupling in the UD model assumes that the magnetostriction coefficient is very small. Hence, the elastic strain is approximately equal to the total strain (${{\boldsymbol{\varepsilon }}}^{tot}\approx {{\boldsymbol{\varepsilon }}}^{el}$). However, when dealing with materials with high magnetostriction constants, the BD and UD models lead to drastically different results.

## Results and Discussions

**Figure 2:**
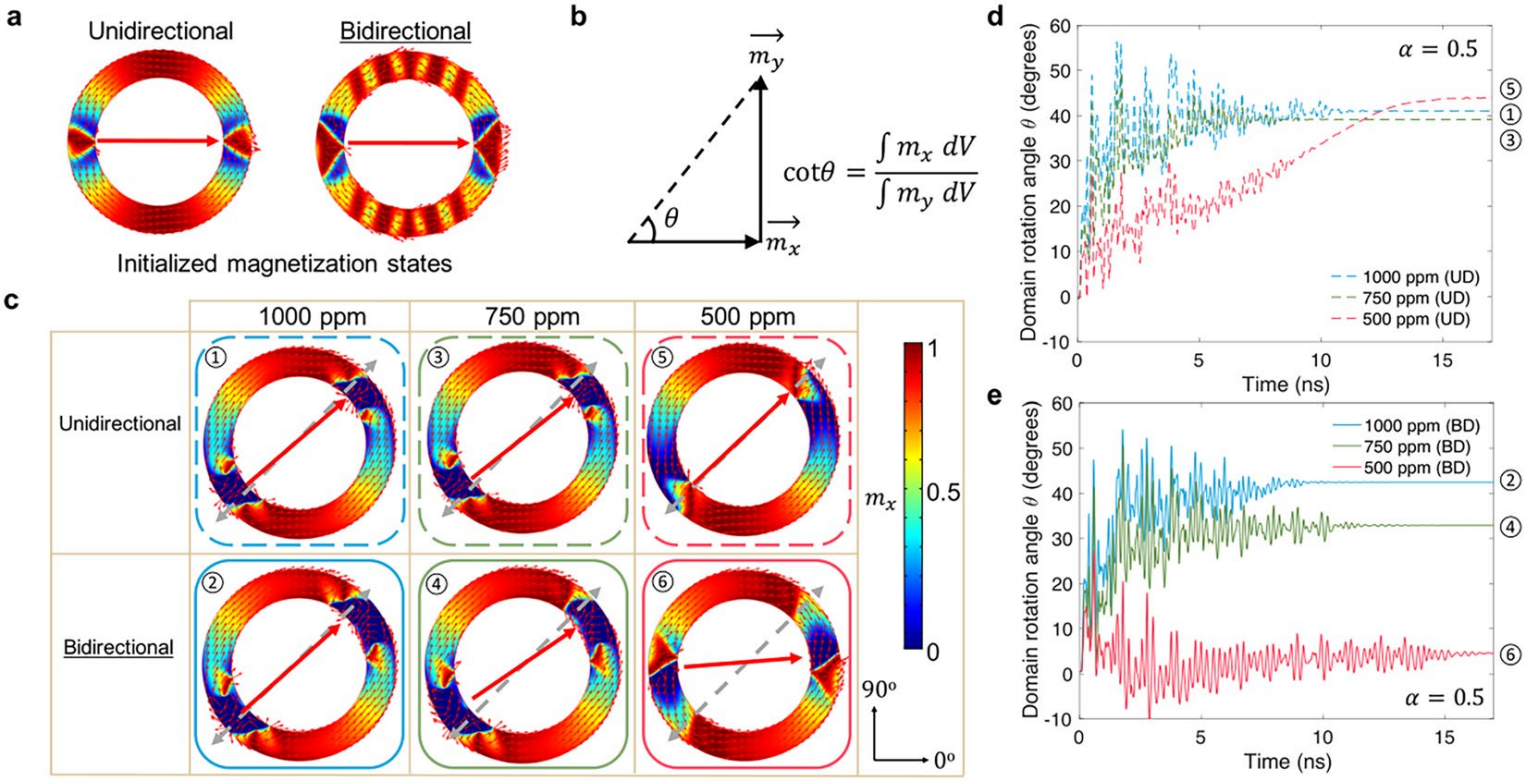
(**a**) Results of finite element simulations for the initialized magnetization state in Terfenol-D ring (*OD* of 1 μm, *w* of 150 nm*, t* of 15 nm) at equilibrium, using unidirectional model (left panel) and bidirectional model (right panel). (**b**) Domain rotation angle is defined as the ratio between the volume average of magnetization along +*x* axis and that along +*y* axis. (**c**) Magnetization distribution and domain rotation state resulting from both models under applied strain of 1000 ppm, 750 ppm and 500 ppm. Solid arrow (in red) defines the final position (after rotation) of the two DWs, while the dashed arrow (in gray) indicates the orientation of the tensile strain. The color gradient bar represents magnetization component along +*x* axis. ①–⑥ are the surface view of stabilized magnetization states after strain is applied. (**d**,**e**) DW rotation angle as a function of time when tensile strain is generated along the direction 45° to the +*x* axis, for UD and BD models, respectively. Simulation time scale is based on damping factor $\alpha$ = 0.5 (time scales are for relative comparison between simulations). The numbers adjacent to the domain rotation angles at equilibrium correspond to the domain state configurations shown in (**c**).

**Figure 3:**
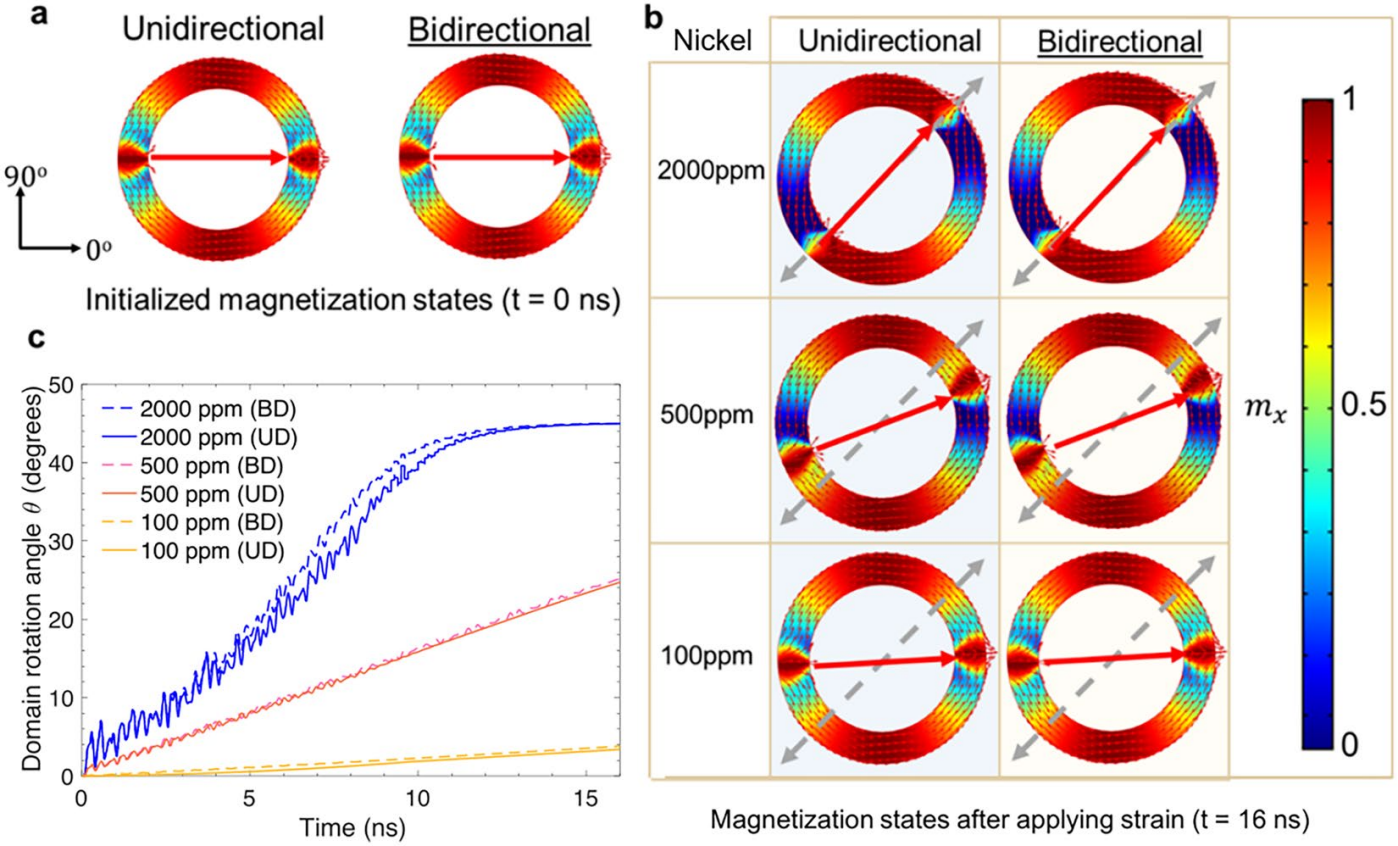
(**a**) Results of finite element simulations of initialized magnetization state of a 15 nm thick, 150 nm wide, 1 μm outer diameter Ni ring at equilibrium. Left panel, result from unidirectional model. Right panel, result from bidirectional model. (**b**) DW rotation state in Ni rings at 16 ns after strain application predicted by two models when subjected to 2000 ppm, 500 ppm and 100 ppm strains. (**c**) DW rotation angle as a function of time as the strain is applied to the piezoelectric substrate at 45° to the +*x* axis (damping factor $\alpha =0.5$ used in simulation), with results from both UD and BD models.

A finite element simulation using micromagnetic/elastodynamic model was developed using COMSOL^43^. The simulation reproduces the initialized “onion” domain state in the Terfenol-D ring as shown in Fig. 2a from unidirectional simulations (left), and from bidirectional simulations (right). Similarly, the initialized magnetization states in Ni rings with the same dimensions can be seen in Fig. 3a.

For a Terfenol-D ring, even though both modeling methods yield an “onion state”, the initial states formed in the simulations based on the UD and BD models are different. The reason for such difference lies in the fact that the FM layer is mechanically-coupled strongly to the FE substrate. For BD modeling, the saturated state initiated to +*x* direction gives more hindrance to the subsequent relaxation due to the strong bi-directional coupling between strain and magetostriction^52^, thus showing “ripple-like” magnetization in the stable onion state. The magnetization direction oscillates periodically along the circumferential direction of the top and bottom side of the ring surface for the bidirectional model. However, no oscillations in the magnetic state are observed from the unidirectional model (see Fig. 2a for comparison). On the contrary, in the less magnetostrictive Ni ring, the two modeling approaches predict almost identical initial states: the magnetization direction exhibits a smoother distribution along the circumferential direction of the ring (see Fig. 3a).

When an electric field is applied to the piezoelectric material, a strain is generated and transmitted to the magnetoelastic material. This strain alters the magnetoelastic energy, causing the onion state to re-orient toward its new energy minimum configuration and driving the DWs towards the direction of the principal strain axes (i.e., compressive directions for Ni and tensile direction for Terfenol-D, respectively). Time-dependent DW rotations are tracked and compared for both UD and BD models. In the case of Terfenol-D rings, we observe in the BD models that feedback from magnetostriction prevents rotation of the DW at lower strain values. The domain rotation angle, $\theta$, is estimated by calculating the ratio of volume averages of the *x*-direction and *y*-direction component of magnetization, as reported in Fig. 2b. As shown in Fig. 2c, the unidirectional model yields full rotation for strain of 1000 ppm (c-1), 750 ppm (c-3), and 500 ppm (c-5). However, the bidirectional model produces a more complex result, where applying a 1000 ppm strain fully rotates the “onion state” to the magnetic easy axis ($\theta$ = 45°, c-2); a 750 ppm strain only partially rotates the domain state to 33° (c-4) and a 500 ppm strain results in minimal rotation of the domain state of 5° (c-6). This is due to the significant influence of the magnetostriction feedback from Terfenol-D rings to the piezoelectric substrate. The time-dependent domain rotation as predicted by both models is shown in Fig. 2d and e. The temporal behaviors of DWs predicted by both models when a strain of 500 ppm is applied are shown in Supporting Videos S1 and S2.

In contrast, for the Ni ring, both modeling approaches give rise to similar DW rotation. due to the small magnetostriction feedback present in Nickel, as shown in Fig. 3b. For Ni, it appears that regardless of the strain magnitude, the DWs fully rotate as long as enough time is given. One explanation is that the low magnetostriction coefficient generates a negligible magnetostrictive effect, which would reduce the initially applied strain.

**Figure 4:**
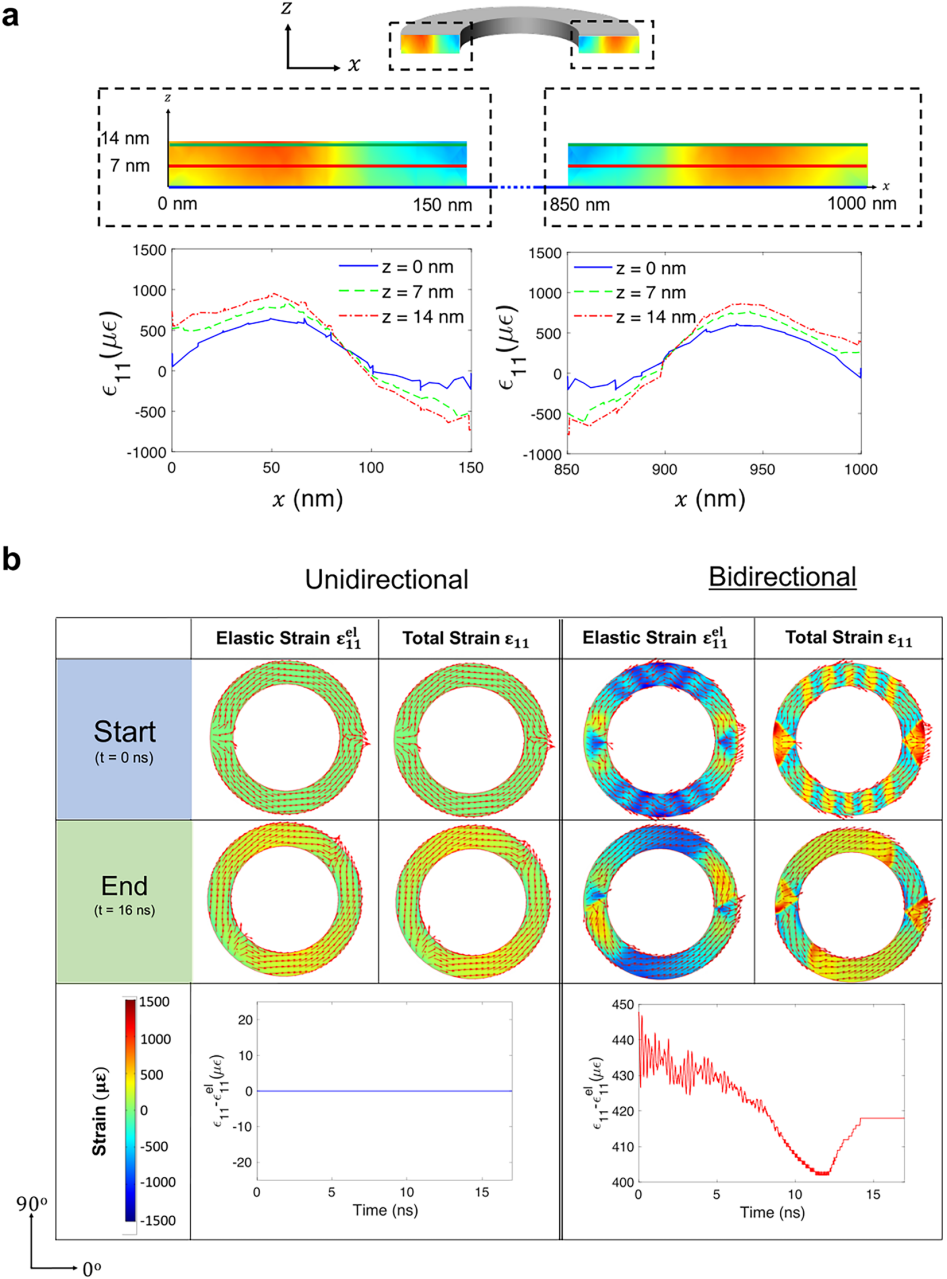
(**a**) Non-uniform strain distribution across the *xz*-plane in the Terfenol-D ring at steady state after applying a strain of 500 ppm using the bidirectional model (top panel) and corresponding strain value as a function of position *x* at three different heights *z* (bottom panel). (**b**) Top view of strain distributions at equilibriums in the ring before and after applying strain (top panel) and difference between total strain and elastic strain as a function of time predicted by two modeling approaches (bottom panel).

To further investigate the difference between these two modeling approaches, we study the size effects in the present microrings. Indeed, we simulate the strain distributions in the ring before and after a strain of 500 ppm is applied to the substrate and transferred to the ring. The non-uniform strain profile in the ring calculated by the bidirectional model is explicitly shown in Fig. 4a. In addition, total strain distributions at different thickness (*z* values) as a function of *x* are plotted to demonstrate the variation of the strain through the thickness of the micro-ring. The difference between total and elastic strain (Fig. 4b), which is zero for UD models and non-zero for BD models, confirms the non-negligible effect of magnetostriction in Terfenol-D. Comparison between the patterns of surface strain mapping plots in the bidirectional model (Fig. 4b) and their corresponding magnetization states (Fig. 2a and c) also indicates the strong coupling between strain and magnetization.

**Figure 5:**
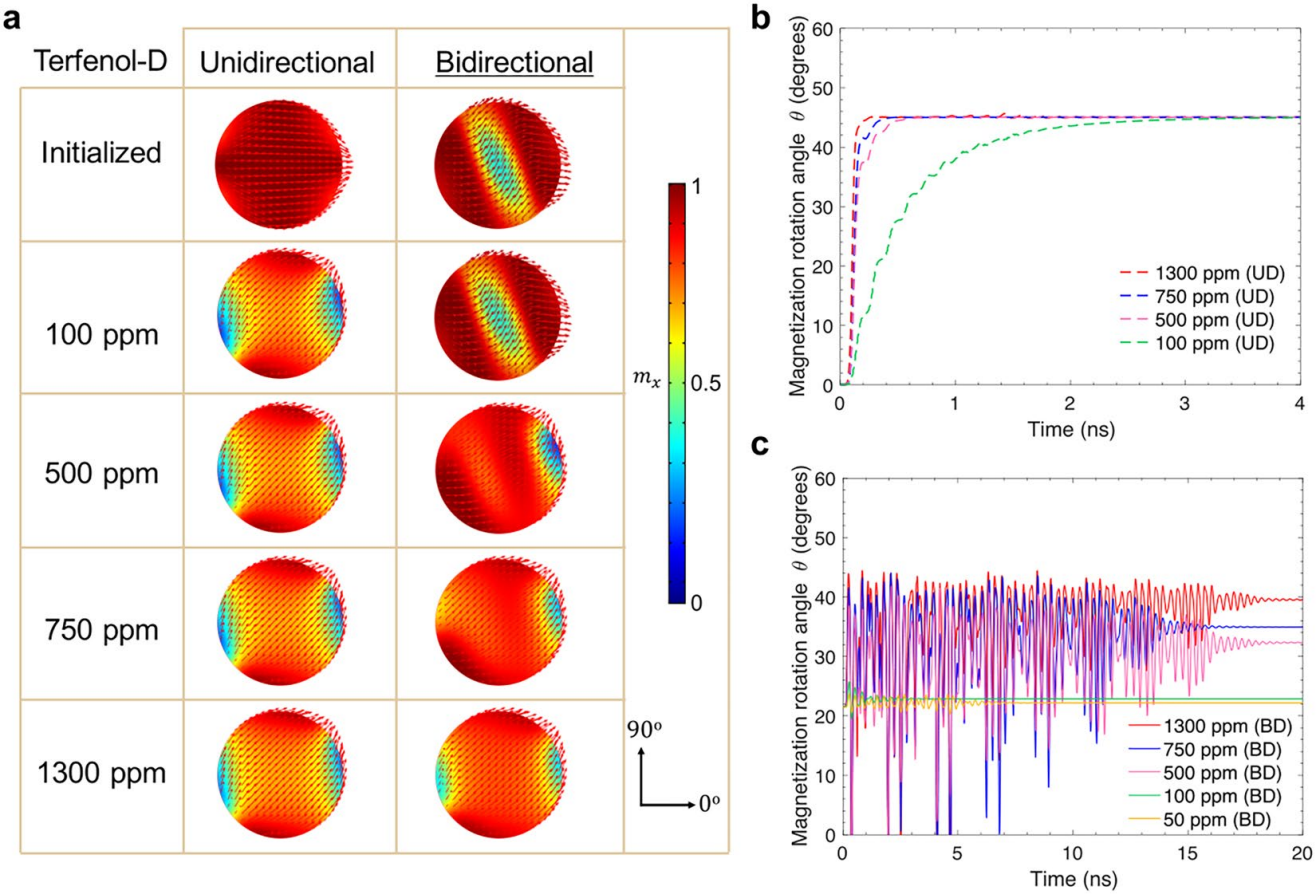
(**a**) Results of finite element simulations of initialized magnetization state of a 15 nm thick, 200 nm diameter Terfenol-D ring at initialization state and at equilibrium after application of strain, predicted by UD and BD models when subjected to 100 ppm, 500 ppm, 750 ppm and 1300 ppm strain. (**b**) and (**c**) Domain state rotation angle as a function of time (damping factor $\alpha =0.5$), obtained by the UD and the BD models, respectively.

**Figure 6:**
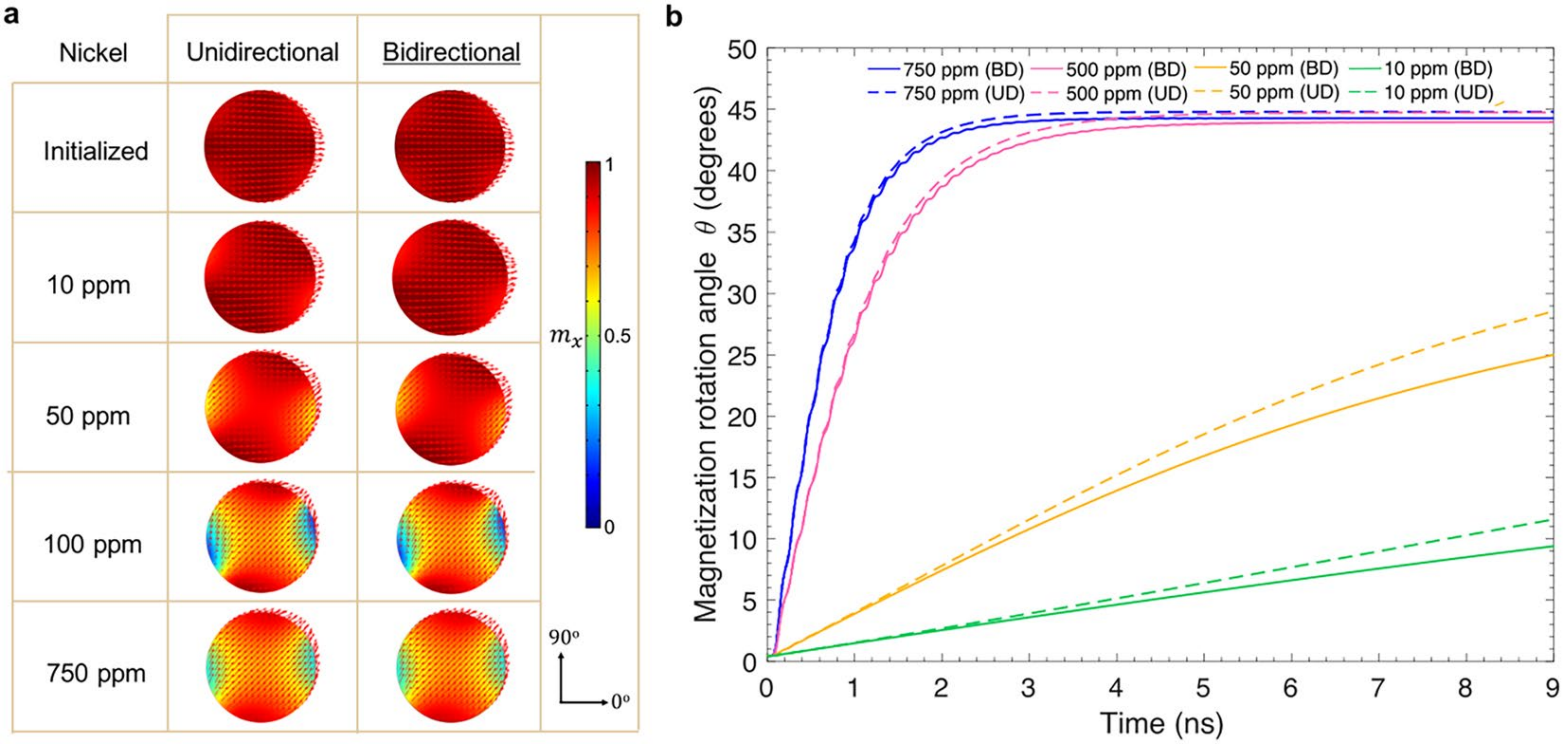
(**a**) Results of finite element simulations of initialized magnetization state of a 15 nm thick, 200 nm diameter Ni ring at initialization state and equilibrium after strain application, predicted by two models when subjected to 10 ppm, 50 ppm, 100 ppm, and 750 ppm strain. (**b**) Domain state rotation angle as a function of time as the strain is applied to the piezoelectric substrate at 45° to the +*x* axis (damping factor $\alpha =0.5$), with results from both UD and BD models.

In order to demonstrate that the importance of the bidirectional modeling approach is not limited only to ring structures, we applied the model to a faster response system, nanometer scale disks, to confirm that the advantage of such model will also be applicable to a wide range of geometries. For a Terfenol-D disk of 200 nm in diameter and 15 nm thick, the equilibrium states at initialization are found to be different. The UD model predicts a single domain state pointing along the initializing magnetic field (+*x* axis, 0°), while the BD model shows an “S” shape domain with average magnetization pointing along 22° (Fig. 5a). The equilibrium magnetic state for Terfenol-D is captured by the BD model, making evident the role of magnetization-induced magnetoelastic strain, ${{\boldsymbol{\varepsilon }}}^{m}$. After applying strain with the BD model, the level of domain rotation in Terfernol-D disk (Fig. 5) is determined by the magnitude of the applied strain. On the contrary, for a Ni disk of the same dimension (Fig. 6), both UD and BD model coincides well with each other, with both yielding a single domain pointing along 0°. When driven by applied strain, the obtained domain state (average magnetization) rotations in the UD and the BD models are equivalent.

The use of BD modeling is most important in cases with highly magnetostrictive materials with low to moderate strain. For weakly magnetoelastic materials, UD and BD models provide similar predictions for strains in the typical range of interest. Whereas for strongly magnetoelastic materials, UD models over predict the rotation of magnetic domains/DWs compared to the more comprehensive BD models. As the strain approaches magnetization saturation $\lambda \approx {\lambda }_{s}$, the predictions of UD and BD models converge. The applied strain is typically smaller than the saturation magnetization (${\lambda }_{s}=1200\times \,{10}^{-6}$ for Terfenol-D), which implies that BD models should generally be used for highly magnetostricive materials. Furthermore, even though the modeling results do not reflect the actual timescale due to selection of $\alpha$, comparison of time-dependent strain rotation plots at different strain levels indicate that the speed of the movement of domains is strain-dependent. With larger applied strain, the domain moves more rapidly.

Finally, it should be noted that the comprehensive bidirectional computational approach here discussed fully captures the magnetic behavior of strain-based multiferroic heterostructures. The unidirectional approach may be helpful for simplifying computation, but does not accurately reflect the behavior of systems in cases where the feedback from magnetization to the strain is comparable to the inverse magnetostrictive effect. This work explains the nuance in coupling between strain and magnetism for the two modeling approaches. The most relevant result is that the two modeling approaches arrive at drastically different predictions in both the equilibrium state and the degree of strain-induced domain rotation. Instead of only leading to a binary result of either full rotation or no rotation as in the case of a unidirectional model, the bidirectional model can produce partial rotation for an intermediate range of applied strains. For the Terfenol-D ring in the bidirectional scenario, strain of 750 ppm does not fully rotate the onion states to 45°, and strain of 500 ppm only minimally rotates the magnetic DWs. The final angle of rotation at equilibrium falls between 0 and 45° depending on the competition between inverse magnetostrictive effect and magnetostrictive effect. Similarly, for the Terfenol-D disk in the bidirectional cases, strains of 750 ppm and below do not fully rotate the domain state due to the additional strain term ${{\boldsymbol{\varepsilon }}}^{m}({\boldsymbol{m}})$. For 1000 ppm strain on ring (Fig. 2c) and 1300 ppm strain on disk (Fig. 5a), the final domain rotation with the UD model is on par with those of the BD model because the strain values are close to Terfenol-D’s magnetostriction coefficient, thus overcoming the energy barriers to reach the maximum rotation state. In other words, the rotation in the UD saturates at a high strain level, and the BD eventually catches up at a higher strain level. It should be noted however, the intermediate states for UD and BD still differ, as evaluated by the rotation angle versus time. Such discrepancy is caused by the large magnetostriction feedback and is therefore more evident in Terfenol-D than in Ni. Therefore, simply by using highly magnetostrictive materials it does not necessarily lead to an enhanced control of magnetism by strain as compared to weakly magnetostrictive materials.

## Conclusions

In summary, we have studied strain-induced domain wall rotation in multiferroic heterostructures using finite element simulations that use both unidirectional and bidirectional coupling approaches. Results from these two types of modeling are compared for strain-induced magnetic-domain rotation in Ni and Terfenol-D rings and disks. The unidirectional approach, while being commonly used by the micromagnetics modeling community, has limitations when evaluating the behavior of highly magnetoelastic materials (e.g. Terfenol-D). The bidirectional model provides a more accurate description of the coupled systems, implying that the effect of magnetostriction feedback from highly magnetoelastic materials to the piezoelectric substrate is non-negligible. The need for bidirectional modeling is highlighted by the fact that the unidirectional model consistently under-predicts the strain necessary to rotate the magnetization in the Terfenol-D rings. Also, the lack of magnetostriction in unidirectional models leads it to predict different initial equilibrium states (before strain is applied) in some cases. Therefore, utilizing bidirectional modeling is key to the understanding of highly efficient multiferroic devices. In addition to the ring and disk shape geometries and Terfenol-D alloy used for demonstration, the fundamental principles in this work are also pertinent to other highly magnetoelastic materials with various in-plane geometries. In combination with experimental investigations, such a bidirectional model may allow for a better assessment of coupling behaviors within multiferroic devices.

## Electronic supplementary material

Supporting information

Supporting Video 1

Supporting Video 2
