# Supplementary material for: Bi-directional coupling in strain-mediated multiferroic heterostructures with magnetic domains and domain wall motion
Source: Sci Rep. 2018 Mar 26;8:5207. doi: 10.1038/s41598-018-23020-2 (PMC5913354; doi:10.1038/s41598-018-23020-2)
Supplement: Supplementary file 1 — Supporting information [file 41598_2018_23020_MOESM1_ESM.pdf]

Supporting Information

Bi-directional coupling in strain-mediated multiferroic heterostructures with magnetic domains and domain wall motion

Zhuyun Xiao, [†] *Roberto Lo Conte,* [§] *Cai Chen,* [‡] *Cheng-Yen Liang,* [‡] *Abdon Sepulveda,* [‡] *Jeffrey Bokor,* [§] *Gregory P. Carman,* [‡] *and Robert N. Candler* [*, †]

†Department of Electrical and Computer Engineering, University of California, Los Angeles, California 90095, USA
§Department of Electrical Engineering and Computer Sciences, University of California, Berkeley, California 94720, USA
‡Department of Mechanical and Aerospace Engineering, University of California, Los Angeles, California 90095, USA
¶California NanoSystems Institute, Los Angeles, California 90095, USA
*rcandler@ucla.edu

Note S1 Initial magnetization classifications

For nanoscale magnetic structures, it is mainly the competition between magnetic anisotropies and the geometry of the structures that determines the initialization state at the equilibrium, which relaxes from the saturated initial state, where all individual magnetic moments throughout the ring point towards +*x*-direction (Figure 1b).[1] During relaxation from the saturated initial state, the competition between exchange energy and magnetostatic energy (i.e., demagnetization energy) plays a pivotal role for total energy minimization in the magnetic structure. The micromagnetic simulation model can be simplified for characterizing the dependence of the initial mapping of domain states on the ring dimensions. For obtaining initial mapping of domain states, we only consider the dominant energy terms, i.e., exchange energy and magnetostatic energy, from the Landau-Lifshitz-Gilbert (LLG) equation.[2] In this way, such initial remanent states for various ring geometries can be approximated using micromagnetics alone.

To find the magnetic ground state in the magnetoelastic rings, we use OOMMF micromagnetic simulator to identify domain states attainable in rings with outer diameter (*OD*)

on micron scale, width (*w*) and thickness (*t*) on submicron scale. According to previous investigation on Ni rings, at relaxation states after initialization, these submicron size scales rings are capable of achieving an "onion state" with either transverse DWs or vortex DWs on opposite sides of the ring, and a vortex state with flux closure domains around the ring.[3] DW states at equilibrium in Terfenol-D rings with different dimensions have been modeled using the OOMMF micromagnetic simulation with an initial magnetization all aligned in +*x* direction.[4]

In this work, we simulated nanoscale rings with a variety of dimensions, including rings with *OD* of 1 μm, *w* of 50 nm, 150 nm, 300 nm, and 400 nm, and *t* of 15 nm, 30 nm and 45 nm, along with rings with *OD* of 2 μm, *w* of 150 nm, 300 nm, 400 nm, 600 nm, and *t* of 15 nm, 30 nm and 45 nm. Correlation between energy density and magnetic configurations in those ring structures is further examined to produce phase diagrams with design specifications for rings with geometries that are energetically favorable for the desired onion states. [2,5]

The relevant length scales for the ring structure are the *OD, w, t* and the magnetostatic exchange length l_{ex} expressed as $l_{ex} = \sqrt{\frac{2A}{\mu_0 M_s^2}}$, where A is the exchange stiffness, and M_s is the saturation magnetization.[5] For Terfenol-D, the calculation is performed based on the following parameters, $A = 1.0 \times 10^{-11}$ J/m and $M_s = 8.0 \times 10^5$ A/m, giving $l_{ex} = 5.0$ nm.[6]

For the micromagnetic model, the maximum ***dm**/dt* is set as 0.02 for a more precise simulation of domain states in Terfenol-D ring structures with *OD* of 1 μm and 2 μm. Due to the competition between demagnetization and exchange energies, the magnetic ring states vary from onion states to vortex state with a flux closure configuration when the ring's width is increased. Onion states with transverse DWs are of interest due to their large energy density and stray field. The energy flux out of the ring can have practical applications in trapping and/or interacting with

nano- and micro-scale particles in the surroundings of the ring via localized magnetostatic interaction.[7] Depending on the ring dimension and *w/OD* ratio, the magnetic energy density of rings with the same *OD* versus thickness of the ring are plotted in Figure S1a-b. Magnetic domain states associated with some of the representative data points are also shown in the plot. Figure S1a shows the magnetic energy density and equilibrium magnetic states after initialization for rings with *OD* of 1 µm and Figure S1b shows these for rings with *OD* of 2 µm. For rings with fixed *OD* of 1 µm and *t* of 45 nm, the increase in ring width leads to DW configuration changing from vortex domain onion state to vortex onion state for magnetic energy minimization of the rings. In the cases with fixed *OD* of 1 µm and *w* of 300 nm, as *t* increases from 15 nm to 30 nm, and 45 nm, domain configuration changes from transverse domain onion state to vortex state, as shown in Figure S1a. Similarly, as shown by the domain configurations in Figure S1b, increase in *t* from 15 nm to 45 nm shows domain state transitioning from transverse domain onion state to vortex domain onion state for Terfenol-D rings with 2 µm in diameter. Calculation of ring energy density and categorization of domain states result in partial phase diagrams for Terfenol-D rings, as shown in Figure S1c-d.[2] The phase diagram which suggests various forms of domain states at equilibrium following initialization, depending on the ring dimensions, is particularly useful for the experimental design of Terfenol-D rings.

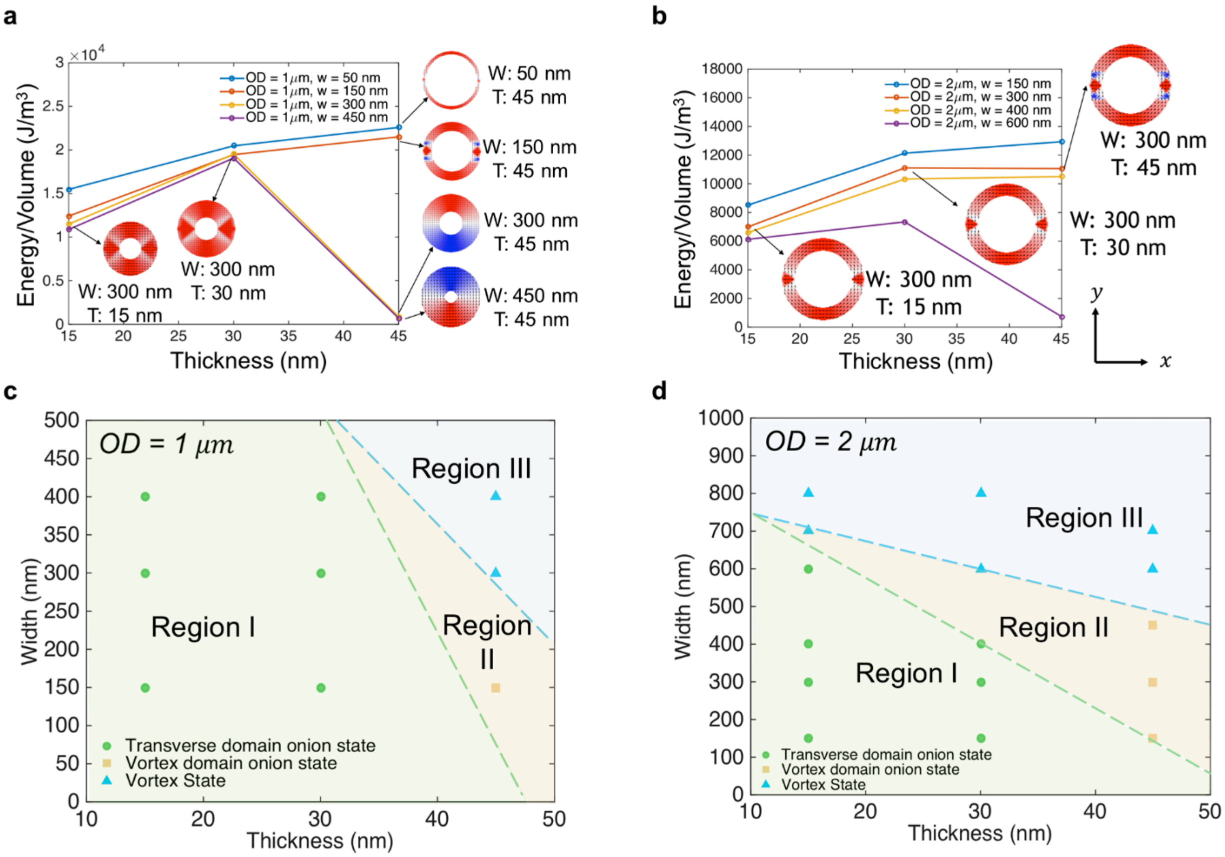

Figure S1. Magnetic energy density (as determined by micromagnetic modeling) vs. Terfenol-D ring's thickness. Each ring structure shows its final magnetic domain state (with spin structure) at equilibrium after initializing all magnetic moments in the $+x$ direction and allowing them to relax to equilibrium. (a) Rings with *OD* of 1 μm. (b) Rings with *OD* of 2 μm. (c)-(d) Calculation of the DW energies gives rise to a proposed phase diagram for DW transition with respect to the ring dimensions (ring width w versus thickness t) for both (c) *OD* of 1 μm rings, and (d) *OD* of 2 μm rings.

Note S2 Equivalent coupled model setup

To reduce simulation time while maintaining accurate results, the multiphysics finite element model can also be set up with a Terfenol-D or Ni ring on top of a SiO_2 substrate, as shown in

Figure S2. This separates the computation required for piezoelectric strain to a separate modeling step. Tensile strain is then induced along 45° in the counter clockwise direction away from the $+x$ axis, and compressive strain along -45° to the $+x$ axis. Therefore, for Terfenol-D with positive magnetostriction, magnetization and DWs tend to orient along 45° away from the $+x$ axis (i.e., along tensile strain direction), same DW behavior as on PMN-PT substrate.

The purpose of choosing a thickness of 500 nm for the substrate rather than a few hundred of microns to match PMN-PT substrate thickness is to make the computations feasible while not affecting the results.

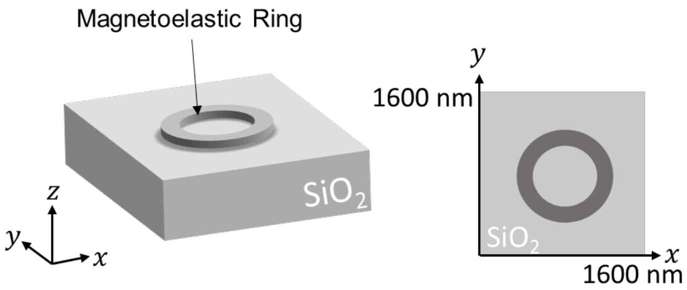

Figure S2 Geometry of the setup in COMSOL Multiphysics, where strain is applied to SiO_2 substrate to achieve similar effect as applying voltage to the piezoelectric substrate.

Note S3 Influence of ramping speed on the domain wall dynamics

The speed of ramping electric field/ strain can affect the domain wall rotation dynamics. Strain of different amplitudes are applied to the substrate with the following ramping slopes k = 10^{10} s^{-1}, 10^9 s^{-1}, and 10^8 s^{-1}, and the corresponding time for ramping t is 0.1 ns, 1 ns and 10 ns. Here, we show the average magnetization angle rotation dynamics predicted by bidirectional model for Terfenol-D ring with an applied strain of 500 ppm (as shown in Figure S3). By comparison, we conclude that the ramping slope of the electric field/strain affects the dynamics of the system, but

can lead to similar magnetization states in the ring at equilibrium following the application of strain for this case.

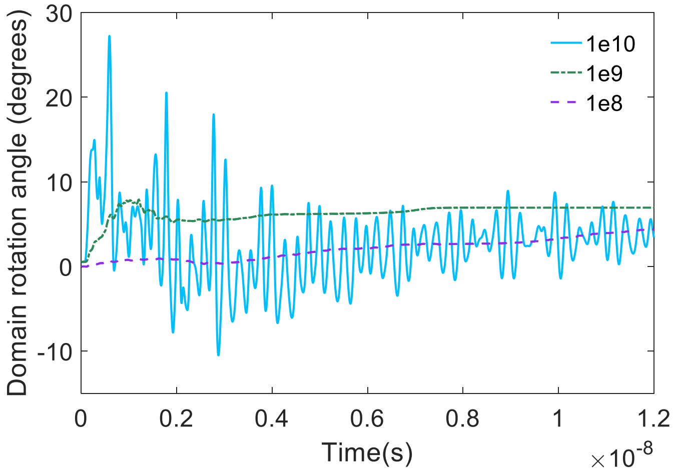

Figure S3 Domain wall angle as a function of strain application time in Terfenol-D rings calculated by bidirectional models, with a strain of 500 ppm generated in the substrate.

Note S4 Influence of mesh size and time step on convergence

To compare the effect of mesh size and time step on convergence, we used the BD model to predict magnetization variation in the Terfenol-D ring when a 1000 ppm strain is applied. The model setup that produces the result in the main text adopts a mesh size of 10 nm for the ring, and 40 nm for the substrate. A model with much finer mesh element setup has a mesh size of 5 nm (close to the exchange length in Terfenol-D) for the ring, and 10 nm for the substrate. The time step is taken as 5 ps for the first model, and 1ps for the second model. As shown in Figure S4, magnetization rotation predicted by the BD model with larger mesh size (S4, left) predicts the same tendency as modeled by the BD model with smaller mesh size (S4, right). With a dramatically increased number of mesh elements, the latter model obtains better convergence

during simulation (convergence error below 10^{-1}), while still following the same domain rotation trend as predicted by the one adopted in the letter. In addition, Figure S5 shows the magnetization configurations in Terfenol-D ring predicted by both models at equilibrium. We thus conclude that the finest mesh size used here is not necessary as it is much more time-consuming and differs little from the ones using larger mesh size. The model used for the letter is sufficient to describe the overall magnetization dynamics in the systems.

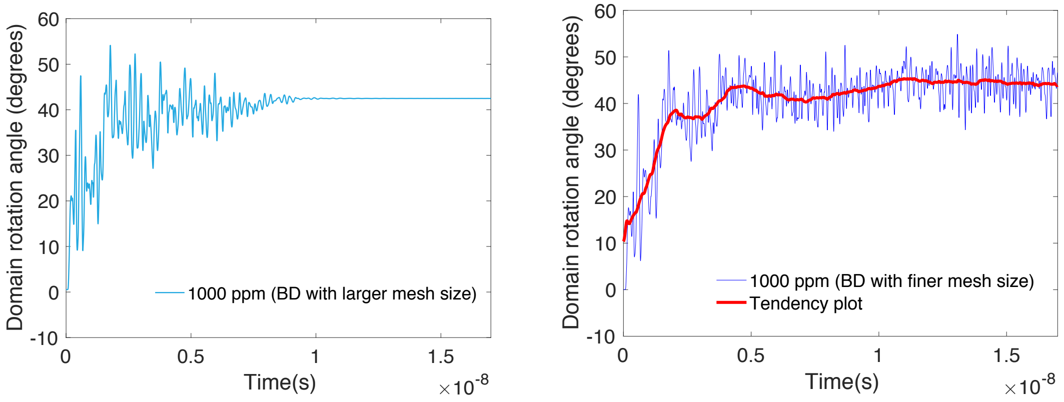

Figure S4 Comparison of domain rotation dynamics predicted by BD model when 1000 ppm strain is applied to the piezoelectric substrate. The plot on the left, shown in the main text of the paper, uses a larger mesh size (10 nm for Terfenol-D, 40 nm for substrate) and a time step of 5 ps. The plot on the right uses a finer mesh size (5 nm for Terfenol-D, 10 nm for substrate) and a time step of 1 ps.

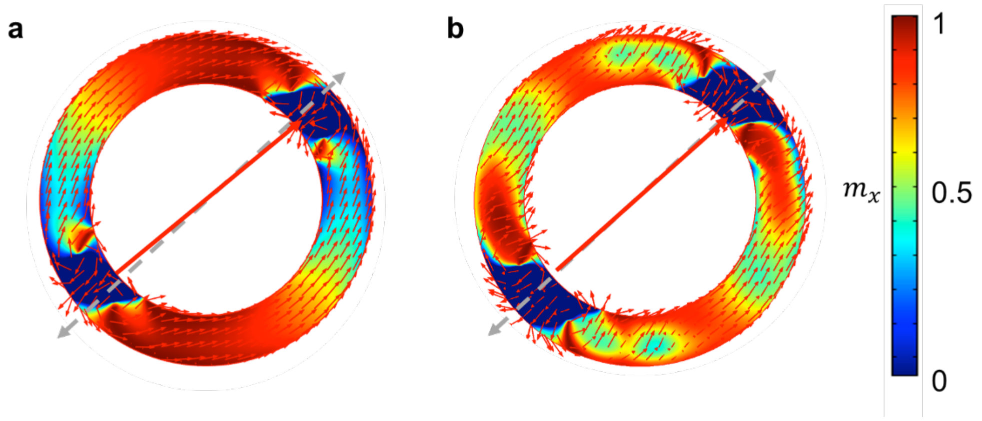

Figure S5 Comparison of the magnetization states in Terfenol-D ring modeled with different mesh sizes at 18 ns. (a) Domain wall rotation angle at 42°, considered as full rotation, predicted by the BD model setup used in the main text of the letter. (b) Domain rotation angle at 42°, considered as full rotation, predicted by the BD model with smaller mesh size, corresponding to Figure S4 (right).

Supporting References

List of Supporting Videos

Supporting Video S1 Domain wall rotation in Terfenol-D ring predicted by a unidirectional model, with an applied strain of 500 ppm (avi format, 1400 frames, 1280*1280px)

Supporting Video S2 Domain wall rotation in Terfenol-D ring predicted by a bidirectional model, with an applied strain of 500 ppm (avi format, 1400 frames, 1280*1280px)

References

1 Streubel, R. *et al.* Magnetism in curved geometries. *Journal of Physics D: Applied Physics* **49**, 363001, doi:10.1088/0022-3727/49/36/363001 (2016).

2 McMichael, R. D. & Donahue, M. J. Head to head domain wall structures in thin magnetic strips. *Ieee Transactions on Magnetics* **33**, 4167-4169, doi:Doi 10.1109/20.619698 (1997).
3 Sohn, H. *et al.* Electrically Driven Magnetic Domain Wall Rotation in :Multiferroic Heterostructures to Manipulate Suspended On-Chip Magnetic Particles. *Acs Nano* **9**, 4814-4826, doi:10.1021/nn5056332 (2015).
4 Donahue, M. J. *OOMMF user's guide [microform] / M.J. Donahue, D.G. Porter*. (U.S. Dept. of Commerce, Technology Administration, National Institute of Standards and Technology, 1999).
5 Ha, J. H., R,. Kirschner, J. . Micromagnetic study of magnetic configurations in submicron permalloy disks. *Physical Review B* **67** (2003).
6 Huang, Y. Y. & Jin, Y. M. Phase field modeling of magnetization processes in growth twinned Terfenol-D crystals. *Appl Phys Lett* **93**, 142504, doi:10.1063/1.2996586 (2008).
7 Rapoport, E., Montana, D. & Beach, G. S. Integrated capture, transport, and magneto-mechanical resonant sensing of superparamagnetic microbeads using magnetic domain walls. *Lab Chip* **12**, 4433-4440, doi:10.1039/c2lc40715a (2012).
